# A cost-effectiveness analysis of lung cancer screening with low-dose computed tomography and a polygenic risk score

**DOI:** 10.1186/s12885-023-11800-7

**Published:** 2024-01-13

**Authors:** Zixuan Zhao, Shuyan Gu, Yi Yang, Weijia Wu, Lingbin Du, Gaoling Wang, Hengjin Dong

**Affiliations:** 1 Department of Public Administration, School of Health Economics and Management, Nanjing University of Chinese Medicine, Nanjing, China; 2 Center for Health Policy and Management Studies, School of Government, Nanjing University, Nanjing, China; 3 Department of Science and Education of the Fourth Affiliated Hospital, and Center for Health Policy Studies, School of Public Health, Zhejiang University School of Medicine, Hangzhou, China; 4 Department of Cancer Prevention, Institute of Cancer and Basic Medicine, Chinese Academy of Sciences/Cancer Hospital of the University of Chinese Academy of Sciences/Zhejiang Cancer Hospital, Hangzhou, China

**Keywords:** Cost-effectiveness, Lung cancer, Polygenic Risk Score, LDCT screening, Markov modelling

## Abstract

**Introduction:**

Several studies have proved that Polygenic Risk Score (PRS) is a potential candidate for realizing precision screening. The effectiveness of low-dose computed tomography (LDCT) screening for lung cancer has been proved to reduce lung cancer specific and overall mortality, but the cost-effectiveness of diverse screening strategies remained unclear.

**Methods:**

The comparative cost-effectiveness analysis used a Markov state-transition model to assess the potential effect and costs of the screening strategies incorporating PRS or not. A hypothetical cohort of 300,000 heavy smokers entered the study at age 50–74 years and were followed up until death or age 79 years. The model was run with a cycle length of 1 year. All the transition probabilities were validated and the performance value of PRS was extracted from published literature. A societal perspective was adopted and cost parameters were derived from databases of local medical insurance bureau. Sensitivity analyses and scenario analyses were conducted.

**Results:**

The strategy incorporating PRS was estimated to obtain an ICER of CNY 156,691.93 to CNY 221,741.84 per QALY gained compared with non-screening with the initial start age range across 50–74 years. The strategy that screened using LDCT alone from 70–74 years annually could obtain an ICER of CNY 80,880.85 per QALY gained, which was the most cost-effective strategy. The introduction of PRS as an extra eligible criteria was associated with making strategies cost-saving but also lose the capability of gaining more LYs compared with LDCT screening alone.

**Conclusion:**

The PRS-based conjunctive screening strategy for lung cancer screening in China was not cost-effective using the willingness-to-pay threshold of 1 time Gross Domestic Product (GDP) per capita, and the optimal screening strategy for lung cancer still remains to be LDCT screening for now. Further optimization of the screening modality can be useful to consider adoption of PRS and prospective evaluation remains a research priority.

**Supplementary Information:**

The online version contains supplementary material available at 10.1186/s12885-023-11800-7.

## Summary

### Evidence before this study

China, with 1/3 proportion of smoking population across the world has substantial cancer burden while lung cancer remains the leading cause of cancer-related death. The effectiveness for mortality reduction of lung cancer screening programs has been well confirmed by several trials (e.g. National Lung Screening Trail) and the challenge for lung cancer screening now seemed to be the high false-positive rate of Low-Dose Computed Tomography (LDCT). To make the existing cancer screening programs more efficient targeting, polygenic risk scores (PRSs) are introduced. PRS have the potential to identify individuals at risk of different type of cancers, optimizing treatment, and predicting survival outcomes. We searched PubMed, EMBASE, and Web of Science between January 1, 2000, and July 30, 2023, with no language restrictions, using the terms “China” or “Chinese”, “lung cancer”, “polygenic risk score” or “PRS” or “genetic test” and “cost-effectiveness”, to identify published economic evaluations on PRS-based strategy for lung cancer screening in China. We found no previous studies describing the cost-effectiveness of PRS-based lung screening in China. Only one previous study evaluated the effect of PRS-based screening based on modelling using UK metrics.

### Added value of this study

The comparative cost-effectiveness analysis used a Markov state-transition model to assess the potential effect and costs of the screening strategies incorporating PRS or not. We found that the screening strategy incorporating PRS was estimated to be cost-effective compared with non-screening, with an ICUR of CNY 156,691.93 to CNY 221,741.84 (initial start age range across 50-74 years) per QALY gained. The strategy that screened using LDCT alone from 70-74 years annually could obtain an ICER of CNY 80,880.85 per QALY gained, which was the most cost-effective strategy. The introduction of PRS as an extra eligible criteria was associated with making strategies cost-saving but also lose the capability of gaining more LYs compared with LDCT screening alone.

### Implications of all the available evidence

Our findings suggest that lung cancer screening programs incorporating PRS of existing performance would hardly be cost-effective using the willingness-to-pay threshold of 1 time GDP per capita, and the optimal screening strategy for lung cancer still remains to be LDCT screening alone for now, suggesting that we should be more conservative in considering LDCT screening with PRS for lung cancer.

## Introduction

Lung cancer is the second most common malignancies in China, where up to 39.8% of all 2.2 million worldwide newly diagnosed cases were from China in 2020 [1, 2]. Only 17.3% of the lung cancer patients are diagnosed at stage I, others are found with advanced stage [3]. Given the large number of patients with lung cancer and the poor prognosis [4], lung cancer contributes prominently to the cancer burden in China with substantial economic and societal impacts in future [5]. To achieve effective cancer prevention, there is a growing focus on improving cancer control through screening and early diagnosis. Several organizations or medical societies worldwide, including National Cancer Center of China, recommended annual low-dose CT (LDCT) screening for people at high risk of developing lung cancer [6–9]. As a result, millions of participants were diagnosed with lung nodules by undergoing LDCT screening every year [10]. However, the false positive rate (FPR) of LDCT test was reported as 96.4% and 56.5% in the National Lung Screening Trial(NLST) and Dutch-Belgian Randomized Lung Cancer Screening Trial(NELSON), respectively [11, 12]. Consequently, a substantial part of subjects undergo unnecessary clinical examinations following a false-positive screening result which results in extra radiation exposure and over-diagnosis.

To make the existing cancer screening programs more efficient targeting, polygenic risk scores (PRSs) are introduced. PRS have the potential to identify individuals at risk of different type of cancers, optimizing treatment, and predicting survival outcomes [13]. Though translation of PRSs into clinically relevant prediction models is a challenge [14, 15]. Recent case–control cohort study suggested that the PRSs could significantly improve discrimination in high risk populations, compared to clinical risk factors (e.g. age, sex, smoking history, cancer histology, etc.) alone [16]. A large-scale prospective cohort study identified 19 susceptibility loci to be significantly associated with non-small cell lung cancer risk at p ≤ 5.0 × 10^−8^,and confirmed that PRS was an independent effective risk stratification indicator beyond age and smoking pack-years in Chinese populations, makes PRS a potential candidate for realizing precision screening [17]. Although promising, none of the candidate PRSs are regularly used in clinical practice, despite studies reporting benefits from using PRS to assess eligibility of several types of cancer screening programs (i.e. breast, prostate and colorectal cancer) [18]. As the PRS could be used as an indicator to guide risk stratification, we propose to use PRS on the basis of former risk assessment criteria to further assess the eligibility of lung cancer screening, might be one of the potential approaches to realize its utility in population-based cancer screening programs. Few results have been reported to date using these PRSs in screening practice; thus, the health outcomes associated with adjunctive strategies with LDCT as well as the cost-effectiveness remain unclear.

Here, we assessed the impact of the current PRS introduced in conjunction with LDCT screening on the effectiveness and cost-effectiveness of lung cancer screening from a societal perspective. Using a Markov model, we evaluated the long-term benefits and harms of lung cancer screening with and without a PRS in Chinese populations.

## Methods

### Study design and model description

**Fig. 1:**
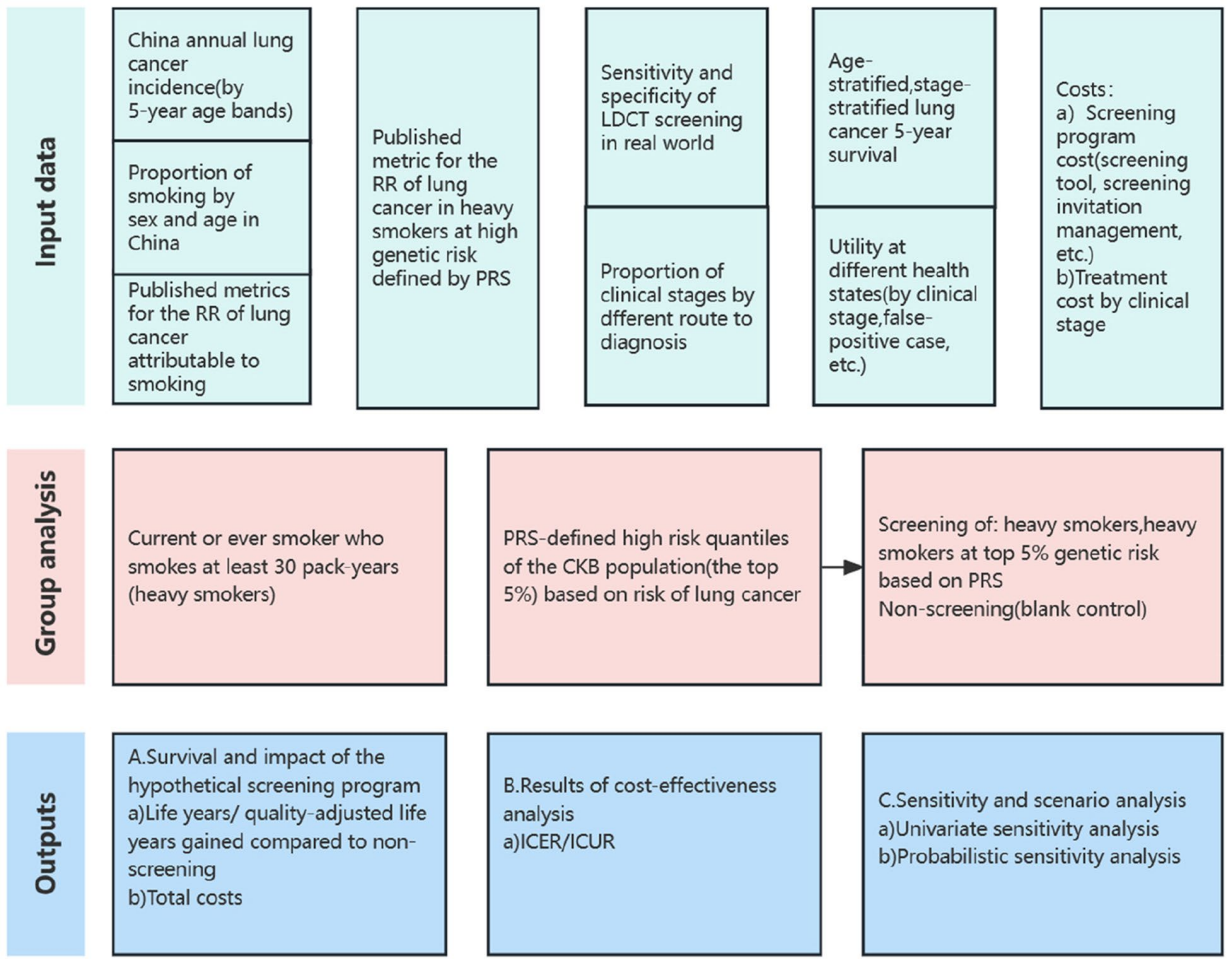
The schematic diagram of research design. Abbreviations: RR, relative risk; LDCT, low-dose computed tomography; PRS, polygenic risk score; CKB, China Kadoorie Biobank; ICER, incremental cost -effectiveness ratio; ICUR, incremental cost-utility ratio

In this modelling study, the Markov model on lung cancer screening that developed by our previous work was used and adapted for the purpose of assessing the potential impact of LDCT screening with and without a PRS from a societal perspective. Important assumptions and the overall structure of the model have been thoroughly described before and in supplementary material [19, 20]. Per China guideline for the screening and early detection of lung cancer (2021,Beijing) [21] recommended, 3 hypothetical cohorts of 10,000 current and former smokers aged 50–74 years old were simulated until death or age 79 years (mean life expectancy in China),named non-screening cohort, LDCT screening cohort and LDCT&PRS screening cohort. Unlike the normal LDCT screening modality, individuals who enter the cohort of LDCT&PRS were assumed to have received PRS assessment and were included to the top 5% high risk based on PRS. All the simulated individuals from two screening cohorts undergo annual screening until the simulation ended. We further superimposed screening and diagnostic follow-up interventions onto the natural history model for lung cancer and obtained population-level outcomes. Data sources, main outcomes, and the full research design are shown in Fig. 1. The model was run with a cycle length of 1 year and a discount rate of 5% was applied to both costs and effectiveness. The model construction and all the simulations were conducted using Treeage Pro, version 2021 (Treeage Software). The study was performed according to the Consolidated Health Economic Evaluation Reporting Standards (CHEERS) and was approved by the ethics committee of the Jiangsu Province Hospital of Chinese Medicine; informed consent was not applicable because this was a modeling study.

### Model input parameters

For this modelling analysis, we used China age-stratified data for lung cancer incidence and integrated the effect of smoking rate to model incidence rates for the initial probability of lung cancer for those in the cohorts of non-screening or LDCT screening alone [22–24]. According to the 3 PRS-defined quantiles (ie, the top 5%,5%-95%, and the bottom 5%), we then calculated the relative risk(RR) of the PRS for lung cancer based on the published estimates of the standardized rates of lung cancer events of the three groups of heavy smokers with diverse genetic risk in China Kadoorie Biobank (CKB) cohort [17]. The proportion of clinical stage for lung cancer detected by LDCT was derived from screening results of the Wenling Lung Cancer Screening Program, which was initiated in 2018 to conduct annual LDCT screening for local populations at high risk of lung cancer with follow-up for 3 years. A total of 20130 asymptomatic individuals were screened by the program by the end of December, 2022, and 287 patients were diagnosed with lung cancer; details of the proportions by cancer stage are presented in Table 1. Annual screening followed the same screening protocol as in the Cancer Screening Program in Urban China, which determined positive findings by morphologic features and the size of the nodule [25]. As for those diagnosed by normal clinical pathways, the probability that diagnosed clinically is detailed by stage in Table 1 based on a hospital-based multi-center lung cancer retrospective clinical epidemiological survey in China(LuCCRES) [26]. The probability of health to all-cause death was estimated as all-cause mortality for smokers by age [24, 27]. The probability of lung cancer-specific death was derived from a study by Zhang et al. [28] and was adjusted for smoking status [29, 30]. The probability that a cancerous state progressed to a more advanced state or to a maintenance state is detailed by cancer stage in Table 1 according to Haaf’s work [31]. The sensitivity and specificity for LDCT were based on a study that enrolled 9,522 person-times over five screening rounds from 2014 to 2018 in Sichuan, China [32]. Perfect attendance to screening was assumed for base-case analysis and the uptake rates by different screening modality were incorporated in scenario analysis [33].

**Table 1:** Input parameters of Markov model

Variables	Base-case value		Distribution	Source
Lung cancer incidence rate in general population(100,000^–1^)	Male	Female		
50–54	84.34	50.87	Beta	[21]
55–59	121.85	56.99	Beta	[21]
60–64	237.82	104.22	Beta	[21]
65–69	329.68	137.74	Beta	[21]
70–74	418.52	178.38	Beta	[21]
RR(> 30 pack-years)	3.87		Lognormal	[34]
RR(> 30 pack-years at top 5% based on PRS)	3.98		Lognormal	[17]
Proportion of lung cancer by stage				Wenling lung cancer screening program
CIS	0.0370		Beta	
I	0.6852		Beta	
II	0.0370		Beta	
III	0.1852		Beta	
IV	0.0556		Beta	
Sensitivity of LDCT	0.79		Beta	[32]
Specificity of LDCT	0.81		Beta	[32]
Mortality of all-cause death (%)				
50–54	0.45		Beta	Estimated [24, 27]
55–59	0.65		Beta	Estimated [24, 27]
60–64	1.08		Beta	Estimated [24, 27]
65–69	1.88		Beta	Estimated [24, 27]
70–74	3.36		Beta	Estimated [24, 27]
75–79	5.40		Beta	Estimated [24, 27]
Lung cancer mortality rate in general population(100,000^–1^)				
50–54	28.81		Beta	[28]
55–59	52.86		Beta	[28]
60–64	101.93		Beta	[28]
65–69	153.34		Beta	[28]
70–74	248.57		Beta	[28]
Transition probabilities(1 year)				
Lung cancer stage CIS to lung cancer stage I	0.0980		Beta	[25]
Lung cancer stage I to lung cancer stage II	0.3682		Beta	[35]
Lung cancer stage I to lung cancer stage III	0.0328		Beta	[35]
Lung cancer stage I to lung cancer stage IV	0.0745		Beta	[35]
Lung cancer stage II to lung cancer stage III	0.2260		Beta	[35]
Lung cancer stage II to lung cancer stage IV	0.1510		Beta	[35]
Lung cancer stage III to lung cancer stage IV	0.1455		Beta	[35]
Lung cancer stage CIS to death	0		Beta	Estimated [28–30]
Lung cancer stage I to death	0.1739		Beta	Estimated [28–30]
Lung cancer stage II to death	0.2842		Beta	Estimated [28–30]
Lung cancer stage III to death	0.4626		Beta	Estimated [28–30]
Lung cancer stage IV to death	0.5880		Beta	Estimated [28–30]
Utility				
CIS	0.92		Beta	[36]
I	0.92		Beta	CanSPUC data
II	0.87		Beta	CanSPUC data
III	0.71		Beta	CanSPUC data
IV	0.60		Beta	CanSPUC data
Maintenance state	0.87		Beta	[37]
Costs(CNY)				Survey data
Screening cost(LDCT)	245.86		Gamma	-
Screening cost(PRS)	280.00		Gamma	-
Pre-diagnosis cost	628.36		Gamma	-
Biopsy diagnosis cost	1,232.44		Gamma	-
Treatment cost				
CIS	47,341.85		Gamma	-
I	53,344.51		Gamma	-
II	83,365.95		Gamma	-
III	90,643.18		Gamma	-
IV	116,471.34		Gamma	-

*Abbreviations: RR* Relative risk ratio, *CIS* Carcinoma in situ, *PRS* Polygenic risk score, *LDCT* Low-dose computed tomography, *CNY* Chinese yuan, *CanSPUC* Cancer Screening Program in Urban China

A total estimated cost for the lung cancer screening program consisted of two parts, the direct screening cost and the indirect screening cost. Screening related cost data were surveyed by the work team of a local lung cancer screening program for the expenses for public advertising, screening invitation management, staff salary and depreciation of screening machinery. For the indirect screening cost, we conducted a survey to estimate the expenses for transportation and wage for missed work for the participants.

We estimated the treatment cost of lung cancer by stage based on the database of local medical insurance bureau, which including 4,947 patients and 107,248 relevant records. Given the potential diversity in treatment cost across the nation, we adapt the treatment cost by stage using published metrics form China Health Statistics Yearbook 2020 [38]. The cost of maintenance by stage was calculated using the standard follow-up process and the unit price of each test per the price list of medical services in public medical institutions. All the costs in this study are expressed in CNY and are discounted to the price level of 2022 at a discount rate of 5%.

For quality-of-life adjustment, we used the utility values for lung cancer state by stage based on a EQ-5D-3L survey from 2586 lung cancer patients in 8 provinces and 12 cities in China through the Cancer Screening Program in Urban China(CanSPUC). In addition to, we derived the utility value of CIS stage from a global systematic review by Sturza et al. [36]. The utility value for the maintenance state of each stage was derived from a domestic thesis in 2016 [37].

### Evaluated strategies

We compared 15 alternative strategies as shown in Table 2. The first 5 strategies involved non-screening for all the heavy smokers as blank control. The remaining 10 strategies were defined by combinations of risk stratification approaches (smoking pack-years or PRS) and initial screening age from 50 to 70 years by 5-year age bands. We describe these strategies in Table 2.

**Table 2:** Characters of the evaluated strategies

Strategy	Eligible criteria	Screening tool	Start age
#0 Non-screening	-	-	50;55;60;65;70
#1 LDCT	Smoking > 30 pack-years	LDCT	50;55;60;65;70
#2 PRS& LDCT	Top 5% based on PRS and smoking > 30 pack-years	LDCT	50;55;60;65;70

*Abbreviations: LDCT* Low-dose computed tomography, *PRS* Polygenic risk score

Pack-years, 1 pack-year equivalent to 20 cigarettes per day for 1 year

### Outcome measures

In this study, primary outcomes included life years (LYs), quality adjusted life years (QALYs), and costs of different strategies. Given the #0 Non-screening strategy as reference, a strategy was deemed cost-effective if the incremental cost-effectiveness ratio (ICER), namely the difference between the overall costs of the two strategies divided by the difference between the total QALYs gained, was lower than the cost-effectiveness threshold of 1–3 times Gross Domestic Production (GDP) per capita per QALY gained (CNY 85,698–257,094) [39].

### Sensitivity analysis and scenario analysis

The robustness of the outcomes to uncertainties in the parameter estimates was examined through a series of univariate sensitivity analyses. The cost of screening, treatment cost as well as maintenance cost and consumer price index (CPI) rate were set to vary by 30% compared to base case values. The discount rate was set to range from 0 to 8%. The RR of the PRS for lung cancer was set to range from 2.64 to 5.99. The sensitivity and specificity of LDCT test were set to range from (0.632, 0.648) to (0.948, 0.972). Furthermore, Probability sensitivity analysis (PSA) was also performed with 10,000 iterations to assess the joint uncertainties in the values of input parameters. Input parameters were randomly drawn from beta, lognormal or gamma distribution (see Table 1). As for the scenario analysis, we evaluated the health benefits and harms associated with a lung cancer screening program that incorporated the uptake rate of different screening modalities among Chinese high-risk population for lung cancer.

#### Software

Modelling was performed in TreeAge Pro 2021 Version R2.1 (TreeAge Software, Williamstown, Massachusetts).

#### IRB approval

This project has been approved by Ethics Committee of the Taizhou cancer hospital (code: IRB-[2020]NO.6).

#### Role of the funding source

No specific funding was received for this analysis.

## Results

### Base-case analysis

In the absence of screening, the total number of lung cancer death per 100,000 heavy smokers aged between 50–79 years were estimated to range from 4,434 to 10,586. The introduction of a screening program led to a decrease of lung cancer deaths, with the reduction rate of lung cancer death ranging from 0.31% to 15.80% across a diverse set of screening strategies. About 95% false-positive cases could be averted by incorporating PRS in the screening program in relative to LDCT screening alone. The LYs and QALYs across all the screening strategies compared with non-screening ranged from 60.26 to 134.93 and from 59.83 to 134.27, respectively. To be specific, screening strategies using PRS as extra eligible criteria obtained lower LY and QALY gained than LDCT screening alone (see Table 3). Compared to non-screening, the #1LDCT strategies cost between CNY 104,998.56 and CNY 176,565.66 per LY gained. The #2 PRS&LDCT strategies cost between CNY 191,110.06 and CNY 260,918.20 per LY gained. When adjusting to QALYs, the #1LDCT strategies would cost between CNY 808,80.85 and CNY 150,050.15 per QALY gained. The #2 PRS&LDCT strategies would cost between CNY 156,691.93 and CNY 221,741.84 per QALY gained. All showed an ICER below 3 times GDP per capita (CNY257,094) per QALY gained. Assuming a cost-effectiveness threshold of 1time GDP per capita (CNY 85,698) per QALY gained for the Chinese healthcare system, only annual LDCT screening with the start age of 65–74 and 70–74 years old were cost-effective, yielding an ICER of CNY 85,332.16 and CNY 80,880.85 per QALY gained compared with non-screening. Table 3 provides the outcomes of the model simulation.

**Table 3:** Outcomes of base-case analysis among alternative strategies

Start age	Strategies	Costs (CNY:million)	LYs (10,000 years)	QALYs (10,000 years)	ICER	ICUR
50	#0 Non-screening	1339.49	134.58	133.86		
	#1 LDCT	1956.54	134.93	134.27	176,565.66	150,050.15
	#2 PRS&LDCT	1386.32	134.60	133.88	260,918.20	221,741.84
55	#0 Non-screening	1283.70	119.71	118.92		
	#1 LDCT	1832.75	120.09	119.38	142,128.81	119,991.71
	#2 PRS&LDCT	1327.11	119.73	118.95	218,831.81	184,753.98
60	#0 Non-screening	1169.75	102.33	101.55		
	#1 LDCT	1634.73	102.73	102.02	116,463.62	97,566.13
	#2 PRS&LDCT	1208.94	102.35	101.57	191,110.06	160,107.15
65	#0 Non-screening	950.81	82.70	82.03		
	#1 LDCT	1309.47	83.04	82.45	104,998.56	85,332.16
	#2 PRS&LDCT	984.64	82.72	82.05	192,795.29	156,691.93
70	#0 Non-screening	631.94	60.26	59.83		
	#1 LDCT	864.37	60.48	60.11	105,370.21	80,880.85
	#2 PRS&LDCT	659.40	60.27	59.84	242,247.42	185,958.48

*Abbreviations: LDCT* Low-dose computed tomography, *PRS* Polygenic risk score, *CNY* Chinese yuan, *LYs* Life years, *QALYs* Quality-adjusted life years, *ICER* Incremental cost-effectiveness ratio, *ICUR* incremental cost-utility ratio

### Sensitivity analysis and scenario analysis

**Fig. 2:**
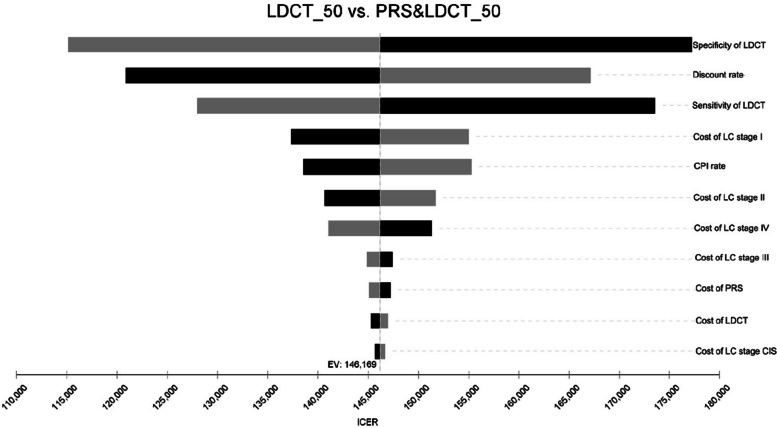
Univariate sensitivity analyses of annual LDCT screening vs PRS&LDCT screening for lung cancer

**Fig. 3:**
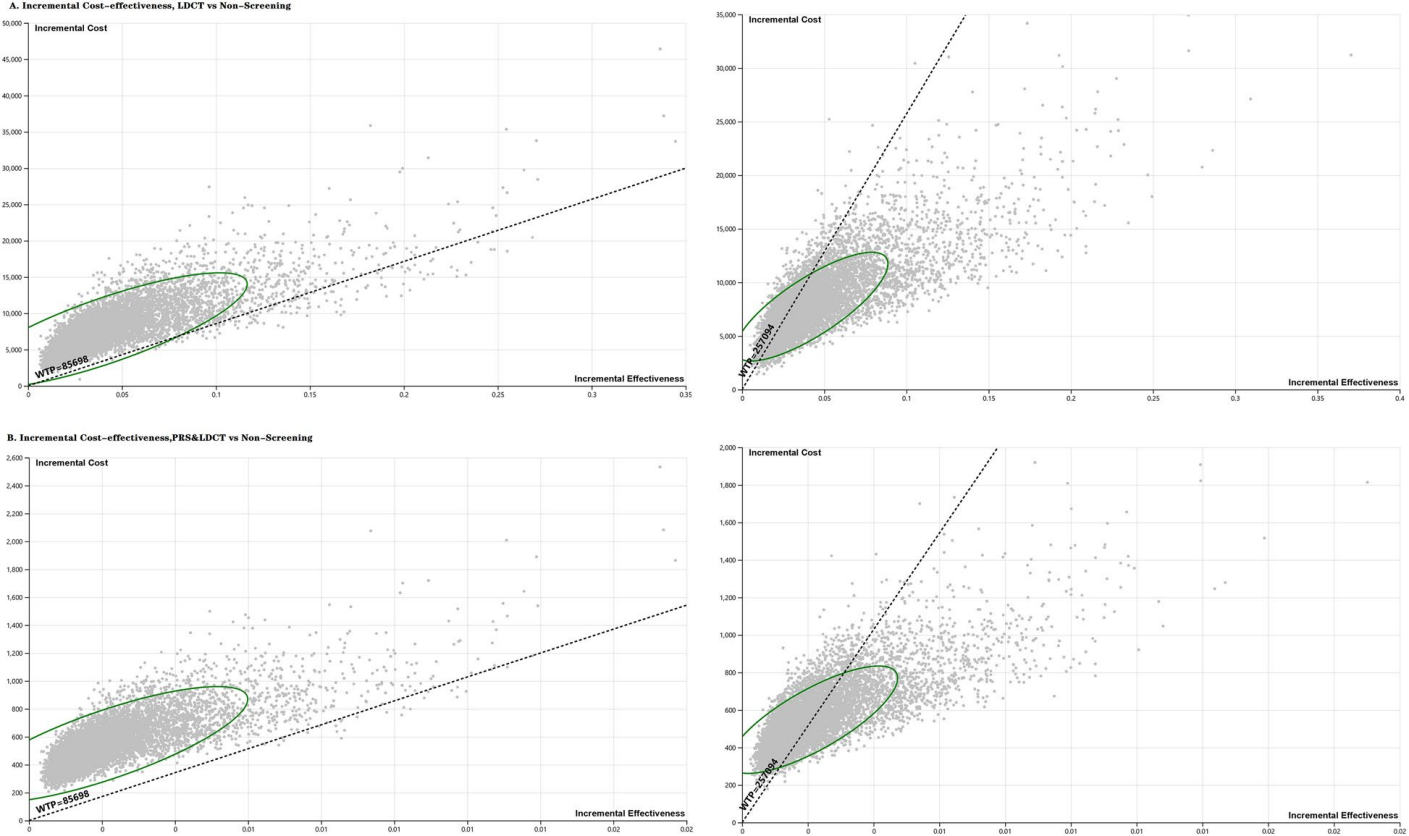
Probabilistic sensitivity analyses of diverse screening strategies for lung cancer

Results of sensitivity analyses are shown in Fig. 2 and Fig. 3. The most influential factors on the ICER were specificity and sensitivity of LDCT, as well as discount rate. The results were robust to the changes of the important values from base-case analysis with no variation exceeding 3 times GDP per capita (CNY257,094) per QALY gained, but also generally exceeding 1 times GDP per capita (CNY85,698) (Fig. 3). Notably, the #1LDCT screening strategy compared with the #0 Non-screening strategy with a start age older than 55 years had better than 90% likelihood of being cost-effective when the willingness-to-pay threshold was 3 times GDP per capita (CNY257,094). Meanwhile, the probability of #2 PRS&LDCT screening strategy to be cost-effective ranged from 33.77%-79.68%, varying from different start age. While the per capita GDP (CNY 85,698) serves as the threshold for absolutely cost-effective, the acceptability at willingness-to-pay threshold ranged from 1.44% to 34.18% for #1LDCT screening strategy and from 0.26% to 2.54% for #2 PRS&LDCT screening strategy (Table 4).

**Table 4:** Acceptability at different level of willingness-to-pay

**Start age**	**Strategies**	**Acceptability at willingness-to-pay (%)**		
		**1 time GDP per capita (CNY85,698)**	**2 times GDP per capita (CNY171,396)**	**3 times GDP per capita (CNY257,094)**
50	#1 LDCT	1.44	27.99	75.07
	#2 PRS&LDCT	0.26	9.89	33.77
55	#1 LDCT	4.31	54.76	93.52
	#2 PRS&LDCT	0.77	16.68	54.34
60	#1 LDCT	10.47	83.35	99.06
	#2 PRS&LDCT	1.88	26.94	73.49
65	#1 LDCT	19.77	94.28	99.81
	#2 PRS&LDCT	2.54	31.9	79.68
70	#1 LDCT	34.18	98.13	99.95
	#2 PRS&LDCT	1.45	22.66	67.41

*Abbreviations*:* LDCT* Low-dose computed tomography, *PRS* Polygenic risk score, *CNY* Chinese yuan, *GDP* Gross Domestic Production

The tornado diagram illustrates the change in the incremental cost-effectiveness ratio (ICER), which was defined as the cost of the PRS&LDCT screening strategy minus the cost of the LDCT screening divided by the difference of the quality-adjusted life-year of the two strategies when important input parameters were varied for both strategies (1 strategy at a time) by 10% ~ 30% higher or lower than their base-case values (shown in Sect. 2.5 Sensitivity analysis and scenario analysis). The vertical axis (dotted dark line) on the left shows the estimated ICER for the base-case analysis, and the vertical axis on the right showed the willingness-to-pay. The column with black color in the tornado diagram showed when the input parameters decrease, their impact for the results. Similarly, the column with grey color showed when the input parameters increase, their impact for the results. 

Abbreviations: LDCT, low-dose computed tomography; PRS, polygenic risk score; LC, lung cancer; CIS, carcinoma in situ; CPI, consumer price index.

The dashed circle is the 95% confidence interval, which indicates the robustness of the model operation. The dashed lines are displayed as the cost-effectiveness threshold of 1 times GDP per capita (CNY85,698) and 3 times GDP per capita (CNY257,094) per QALY gained, respectively. The dots above the dashed line are cost-effective.

Abbreviations: LDCT, low-dose computed tomography; PRS, polygenic risk score; WTP, willingness-to-pay threshold. In a previous study, a discrete choice experiment was used to create scenarios on several different possible modalities for the implementation of lung cancer screening in Chinese context [38]. The uptake rate varied from different screening modalities by mixed-logit model. The uptake rate of screening by blood test would be decreased by 0.08 compared with the baseline, i.e. LDCT screening. The compliance rate of LDCT screening in CanSPUC from 2013 to 2018 remained 34.41%, 37.25%, and 48.21% in urban areas of Shanxi, Henan, and Zhejiang Provinces, respectively [5–7]. However, we found a substantial improvement (91%) on the compliance rate of LDCT in Wenling lung cancer screening program than those reported by CanSPUC. As CanSUPC was a national cancer screening program targeting five cancer types (lung cancer, female breast cancer, liver cancer, upper gastrointestinal cancer, and colorectal cancer) using a combined screening modality. Given the effect on the compliance rate for the combined screening modality of five cancer type might varied from separate screening for each cancer type, we hence used the compliance rate of LDCT screening from the Wenling lung cancer screening program in this study. The compliance rate of PRS test was then estimated as 83.72% for scenario analysis. When we analysed the impact of the compliance rate of LDCT and PRS test, we observed similar patterns as those obtained from our base case analysis with a perfect attendance, despite some differences on the absolute effects due to discrepancies in the compliance rate of the two cohorts (Supplementary Table S6).

## Discussion

We assessed the effectiveness and cost-effectiveness of lung cancer screening per the NCC recommendation when PRS is introduced to further assess the eligibility of lung cancer screening on the basis of the current definitions of high-risk population for lung cancer in China. The results showed that lung cancer screening programs incorporating PRS of current performance would be cost-effective with the start age of 50–74 years, using a willingness-to-pay threshold of 3 times GDP per capita (CNY257,094) per QALY gained. We demonstrated that as the compliance rate of the screening test decreased by 10%-20% (i.e. a real-world like scenario), its start age must be postponed to 55 years for the screening program to be cost-effective. However, when applied the willingness-to-pay threshold of 1 time GDP per capita (CNY85,698) per QALY gained, all the screening strategies incorporating PRS were not able to be cost-effective anymore. Note that the #1LDCT screening strategy were more cost-effective than #2 PRS&LDCT screening strategy using existing PRS tool in general, yielding more LYs or QALYs at lower cost. These results were sensitive to the sensitivity and the specificity of LDCT, as well as the discount rate. The results were robust when incorporating real-world compliance rate of the LDCT and PRS test in place of the perfect attendance. Overall, our results suggested that we should be more conservative in considering LDCT screening with PRS for lung cancer, unless optimized PRS with better performance emerged.

In a modelling study, the Huntley et al. modelled the application of PRS stratification using UK metrics and demonstrated that the PRS-defined high-risk quintile (20%) of the UK population was estimated to capture 26% of lung cancer cases [18]. However, lung cancer was not presented as being the most plausible use cases for PRS stratification on account of the current PRS predictiveness and the availability of established cancer screening tools than other cancer types like breast, prostate, or colorectal cancer [18]. Furthermore, rather than considering age and PRS as mutually exclusive options, it is more rational to consider stratification based on a combination of age and PRS, and the other risk factors (notably, for lung cancer, smoking pack-years and family history) [40].

Nevertheless, a downside of our study is that the modeled strategies cover only one possible group at high risk, i.e. the top 5% based on PRS in the CKB cohort. Due to the crucial effect of smoking status for lung cancer incidence, we were not able to reliably estimate the actual ability to capture lung cancer cases using the area under the receiver operating characteristic curve for PRS alone, nor assess the effect and cost-effectiveness of the scenarios incorporating diverse PRS-defined high-risk quantiles. Hence, there is still a need to further assess the alternative strategies by generating empirical evidence on the utility of risk stratification in population-based screening programs in future. Furthermore, as histologic type was also determinant of long-term outcomes of lung cancer patients, the application for the average probability in the transition probabilities between cancerous states might affect the analytical precision in this work. Further research may benefit from incorporating the histology data for the construction of natural history model for lung cancer.

By introduction of new PRS-stratified screening tool, the application in cancer screening could be considered from diverse perspectives. For mass screening based on population, Huntley et al. focused on providing additional screening to the PRS-defined high-risk group [18], this study explored the modality that adding PRS to the former high-risk criteria to assess eligibility of lung cancer screening. Conversely, using PRS-stratified screening tool to provide less intensive screening to low-risk individuals could also help to reduce the unnecessary harms (i.e. radiation exposure or invasive biopsy) and costs of overscreening. Moreover, several studies have shown that the risk-stratified screening programs [41, 42] and personalized screening randomised trials for breast cancer [43, 44] were ongoing in the Europe and the United States. The risk-tailored screening modality which determine the screening age range, frequency, and method to each risk group according to the PRS might be a potential solution for lung cancer screening programs as well.

Research into new application of PRS in screening programs typically involves breast cancer [45, 46], prostate cancer [47, 48] and colorectal cancer [49, 50]. Current findings can be informative for researchers in the field of cancer epidemiology to guide early adoption of PRS in screening programs or trials for lung cancer, given that they provide extensive information on expected costs, effects, and even cost-effectiveness based on current status. According to our findings, the field of cancer screening and early-detection could move into a direction where PRS will become cost-effective as a molecular diagnostic test in participants with high risk of lung cancer. Although currently the #1LDCT screening strategy were more cost-effective than #2 PRS&LDCT screening strategy using existing PRS tool in general, the obtained data could then potentially be used for a better stratification leading to more participants receiving better screening service. By the time real-world data relevant to the modeled scenarios become available, a more comprehensive and precise cost-effectiveness analysis should be performed for validation purposes. In light of the uncertainties and insufficient performance of the current modality, it seems advisable to accompany adoption with further research to optimize the performance by risk assessment and tailoring of screening frequency and age range of screening for lung cancer.

Our findings suggest that lung cancer screening programs incorporating PRS of current performance would hardly be cost-effective using the willingness-to-pay threshold of 1 time GDP per capita, and the optimal screening strategy for lung cancer still remains to be LDCT screening alone for now. Further optimization of the screening modality can be useful to consider early adoption of PRS, in order to identify the best ways to implement lung cancer screening programs that could improve the benefit–harm trade-offs and cost-effectiveness relevant to its implementation.

### Supplementary Information

**Additional file 1. Appendix 1. **Operational validation for the natural history model of lung cancer. **Figure S1.** Schematic diagram of Markov model for lung cancer screening. **Table S1.** Initial and death probability of natural history model. **Table S2.** Transition probabilities in natural history model for lung cancer. **Table S3.** Validity indicators and sources. **Table S4.** Standard population of China and Segi’s population. **Table S5.** Validity indicator: incidence and mortality of lung cancer. **Figure S2.** Proportion for clinical stages. **Figure S3.** Comparison between GBD observed value and simulation value in life expectancy. **Appendix 2.** Scenario analysis. **Table S6.** Outcomes of scenario analysis with diverse compliance rates. **Appendix 3.** CHEERS Checklist.

## References

[1] Sung H, Ferlay J, Siegel RL (2021). Global cancer statistics 2020: GLOBOCAN estimates of incidence and mortality worldwide for 36 cancers in 185 countries. CA Cancer J Clin.

[2] Ferlay J, Ervik M, Lam F, et al. Global cancer observatory: cancer today. lyon, france: international agency for research on cancer. Available from: https://gco.iarc.fr/today. Accessed 29 Nov 2022.

[3] Zeng H, Ran X, An L (2021). Disparities in stage at diagnosis for five common cancers in China: a multicentre, hospital-based, observational study. Lancet Public Health.

[4] SEER Cancer Stat Facts: Lung and Bronchus Cancer. National cancer institute. https://seer.cancer.gov/statfacts/html/lungb.html. Accessed 15 June 2023.

[5] Zang S, Zhan H, Zhou LR (2019). Research on current curative expenditure among lung cancer patients based on the "System of Health Accounts 2011": insights into influencing factors. J Cancer.

[6] He J, Li N, Chen W (2021). China Guideline for the Screening and Early Detection of Lung Cancer (2021, Beijing). Chinese Journal of Oncology.

[7] Krist AH, Davidson KW, Mangione CM (2021). Screening for Lung Cancer: US Preventive Services Task Force Recommendation Statement. JAMA.

[8] Mazzone PJ, Silvestri GA, Souter LH (2021). Screening for Lung Cancer: CHEST Guideline and Expert Panel Report. Chest.

[9] Oudkerk M, Devaraj A, Vliegenthart R (2017). European position statement on lung cancer screening. Lancet Oncol.

[10] Horeweg N, Scholten ET, de Jong PA (2014). Detection of lung cancer through low-dose CT screening (NELSON): a prespecified analysis of screening test performance and interval cancers. Lancet Oncol.

[11] Aberle DR, Adams AM, Berg CD (2011). Reduced lung-cancer mortality with low-dose computed tomographic screening. N Engl J Med.

[12] de Koning HJ, van der Aalst CM, de Jong PA (2020). Reduced Lung-Cancer Mortality with Volume CT Screening in a Randomized Trial. N Engl J Med.

[13] Klein RJ, Gümüş ZH (2022). Are polygenic risk scores ready for the cancer clinic?—a perspective. Transl Lung Cancer Res.

[14] Ala-Korpela M, Holmes MV (2020). Polygenic risk scores and the prediction of common diseases. Int J Epidemiol.

[15] Sud A, Turnbull C, Houlston R (2021). Will polygenic risk scores for cancer ever be clinically useful?. NPJ Precis Oncol.

[16] Mikey B. Lebrett, Miriam J. Smith, Emma J. Crosbie, et al. Validation of lung cancer polygenic risk scores in a high-risk case-control cohort. Genet Med. 2023:100882. 10.1016/j.gim.2023.100882. 10.1016/j.gim.2023.100882 37154150

[17] Dai J, Lv J, Zhu M (2019). Identification of risk loci and a polygenic risk score for lung cancer: a large-scale prospective cohort study in Chinese populations. Lancet Respir Med.

[18] Huntley C, Torr B, Sud A (2023). Utility of polygenic risk scores in UK cancer screening: a modelling analysis. Lancet Oncol.

[19] Zhao Z, Wang Y, Wu W (2022). Cost-effectiveness of low-dose computed tomography with a plasma-based biomarker for lung cancer screening in China. JAMA Netw Open.

[20] Zhao Z, Du L, Li Y (2022). Cost-effectiveness of lung cancer screening using low-dose computed tomography based on start age and interval in China: modeling study. JMIR Public Health Surveill.

[21] He J, Li N,Chen W,et al. China guideline for the screening and early detection of lung cancer (2021,Beijing). Chin J Oncol. 2021;43(3):243–268:193–207. 10.3760/cma.j.cn112152-20210119-00060 10.3760/cma.j.cn112152-20210119-00060 33752304

[22] He J, Chen W. China cancer registry annual report 2018. Beijing: People’s Medical Publishing House; 2019.

[23] Yuan J, Sun Y, Wang K, et al. Cost effectiveness of lung cancer screening with low-dose CT in heavy smokers in China. Cancer Prev Res (Phila). Published online September 27, 2021. 10.1158/1940-6207.CAPR-21-0155. 10.1158/1940-6207.CAPR-21-0155 34580085

[24] Tabulation on the 2010 Population Census of the People’s Republic of China. Department of population and employment statistics. Beijing: National Bureau of Statistics of China; 2010. https://www.stats.gov.cn/sj/ndsj/2010/left.htm.

[25] Chen WQ, Li N, Cao MM (2020). Preliminary analysis of cancer screening program in urban China from 2013 to 2017. China Cancer.

[26] Shi JF, Wang L, Wu N (2019). Clinical characteristics and medical service utilization of lung cancer in China, 2005–2014: Overall design and results from a multicenter retrospective epidemiologic survey. Lung Cancer.

[27] Liu BQ, Peto R, Chen ZM (1998). Emerging tobacco hazards in China: 1. Retrospective proportional mortality study of one million deaths. BMJ.

[28] Zhang M, Chunxiao W, Yangming G (2017). Survival analysis of patients with lung cancer in Shanghai. China Oncology.

[29] Wang DZ, Zhang H, Zhang Y (2012). A population-based case-control study on the relationship between smoking and lung cancer death. J Tuberculosis Lung Health.

[30] Hong S, Mok Y, Jeon C, Jee SH, Samet JM (2016). Tuberculosis, smoking and risk for lung cancer incidence and mortality. Int J Cancer.

[31] Kevin ten Haaf, Joost van Rosmalen and Harry J. de Koning. Lung cancer detectability by test, histology, stage, and gender: Estimates from the NLST and the PLCO trials. Cancer Epidemiol Biomarkers Prev, 2015 (24) (1) 154–161. 10.1158/1055-9965. 10.1158/1055-9965.EPI-14-0745 PMC4357842 25312998

[32] Sozzi G, Boeri M, Rossi M (2014). Clinical utility of a plasma-based miRNA signature classifier within computed tomography lung cancer screening: a correlative MILD trial study. J Clin Oncol.

[33] Zhao Z, Du L, Wang L, Wang Y, Yang Y, Dong H (2021). preferred lung cancer screening modalities in China: a discrete choice experiment. Cancers.

[34] Sun C, Zhang X, Guo S (2021). Determining cost-effectiveness of lung cancer screening in urban Chinese populations using a state-transition Markov model. BMJ Open.

[35] Hofer F, Kauczor HU, Stargardt T (2018). Cost-utility analysis of a potential lung cancer screening program for a high-risk population in Germany: a modelling approach. Lung Cancer.

[36] Sturza J (2010). A review and meta-analysis of utility values for lung cancer. Med Decis Making.

[37] Chen Shuting. A study on the economic burden and quality of life of lung cancer patients. Anhui Medical University; 2016. 10.7666/d.D01025690.

[38] National Health Commission. China Health Statistics Yearbook 2020. Beijing: China Union Medical College Press; 2020. 10, 115. ISBN: 9787567915619.

[39] Iino H, Hashiguchi M, Hori S (2022). Estimating the range of incremental cost-effectiveness thresholds for healthcare based on willingness to pay and GDP per capita: a systematic review. PLoS ONE.

[40] Pashayan N, Easton DF, Michailidou K (2023). Polygenic risk scores in cancer screening: a glass half full or half empty?. Lancet Oncol.

[41] Brooks JD, Nabi HH, Andrulis IL (2021). Personalized risk assessment for prevention and early detection of breast cancer: integration and implementation (PERSPECTIVE I&I). J Pers Med.

[42] McWilliams L, Evans DG, Payne K (2022). Implementing risk-stratified breast screening in England: an agenda setting meeting. Cancers (Basel).

[43] Roux A, Cholerton R, Sicsic J (2022). Study protocol comparing the ethical, psychological and socio-economic impact of personalised breast cancer screening to that of standard screening in the "My Personal Breast Screening" (MyPeBS) randomised clinical trial. BMC Cancer.

[44] Shieh Y, Eklund M, Madlensky L, et al. Breast cancer screening in the precision medicine era: risk-based screening in a population-based trial. J Natl Cancer Inst. 2017;109(5). 10.1093/jnci/djw290. 10.1093/jnci/djw290 28130475

[45] Jeroen J, Clyde B, Nicolien T (2021). Personalizing breast cancer screening based on polygenic risk and family history. J Natl Cancer Inst.

[46] Lee A, Mavaddat N, Wilcox AN (2019). BOADICEA: a comprehensive breast cancer risk prediction model incorporating genetic and nongenetic risk factors. Genet Med.

[47] Callender T, Emberton M, Morris S (2021). Benefit, harm, and cost-effectiveness associated with magnetic resonance imaging before biopsy in age-based and risk-stratified screening for prostate cancer. JAMA Netw Open.

[48] Keeney E, Sanghera S, Martin RM (2022). Cost-effectiveness analysis of prostate cancer screening in the UK: a decision model analysis based on the CAP trial. Pharmacoeconomics.

[49] Cenin DR, Naber SK, de Weerdt AC (2020). Cost-effectiveness of personalized screening for colorectal cancer based on polygenic risk and family history. Cancer Epidemiol Biomarkers Prev.

[50] Dixon P, Keeney E, Taylor JC, Wordsworth S, Martin RM (2022). Can polygenic risk scores contribute to cost-effective cancer screening? A systematic review. Genet Med.

