# Supplementary material for: A cost-effectiveness analysis of lung cancer screening with low-dose computed tomography and a polygenic risk score
Source: BMC Cancer. 2024 Jan 13;24:73. doi: 10.1186/s12885-023-11800-7 (PMC10787978; doi:10.1186/s12885-023-11800-7)
Supplement: Supplementary file 1 — Additional file 1. Appendix 1. Operational validation for the natural history model of lung cancer. Figure S1. Schematic diagram of Markov model for lung cancer screening. Table S1. Initial and death probability of natural history model. Table S2. Transition probabilities in natural history model for lung cancer. Table S3. Validity indicators and sources. Table S4. Standard population of China and Segi’s population. Table S5. Validity indicator: incidence and mortality of lung cancer. Figure S2. Proportion for clinical stages. Figure S3. Comparison between GBD observed value and simulation value in life expectancy. Appendix 2. Scenario analysis. Table S6. Outcomes of scenario analysis with diverse compliance rates. Appendix 3. CHEERS Checklist. [file 12885_2023_11800_MOESM1_ESM.docx]

**Supplementary appendix**

**Appendix 1. Operational validation for the natural history model of lung cancer**

The natural history model of lung cancer simulates the occurrence and development process of cancer in the real world, but in fact, the occurrence and development process of cancer is very complicated, and the accurate natural history process is often difficult to be fully understood. Therefore, there are many conjectures and assumptions about the natural history process when building the model, and the output results of the model have uncertainties. The evaluation of the operational validity of the natural history model thus is of great significance. Lung cancer is assumed to progress sequentially from less advanced to more advanced preclinical stages, as depicted in Fig. S1. Five stages are distinguished based on the American Joint Committee on Cancer (AJCC) Cancer Staging Manual, 8th edition: carcinoma in situ(CIS) I, II, III, and IV. Stages IA, IB and IIIA, IIIB were not considered in this edition. Stage I and III were not divided into stages IA, IB and IIIA, IIIB because data for these stages were not available for Chinese clinical practice or the extracted cancer registry data.


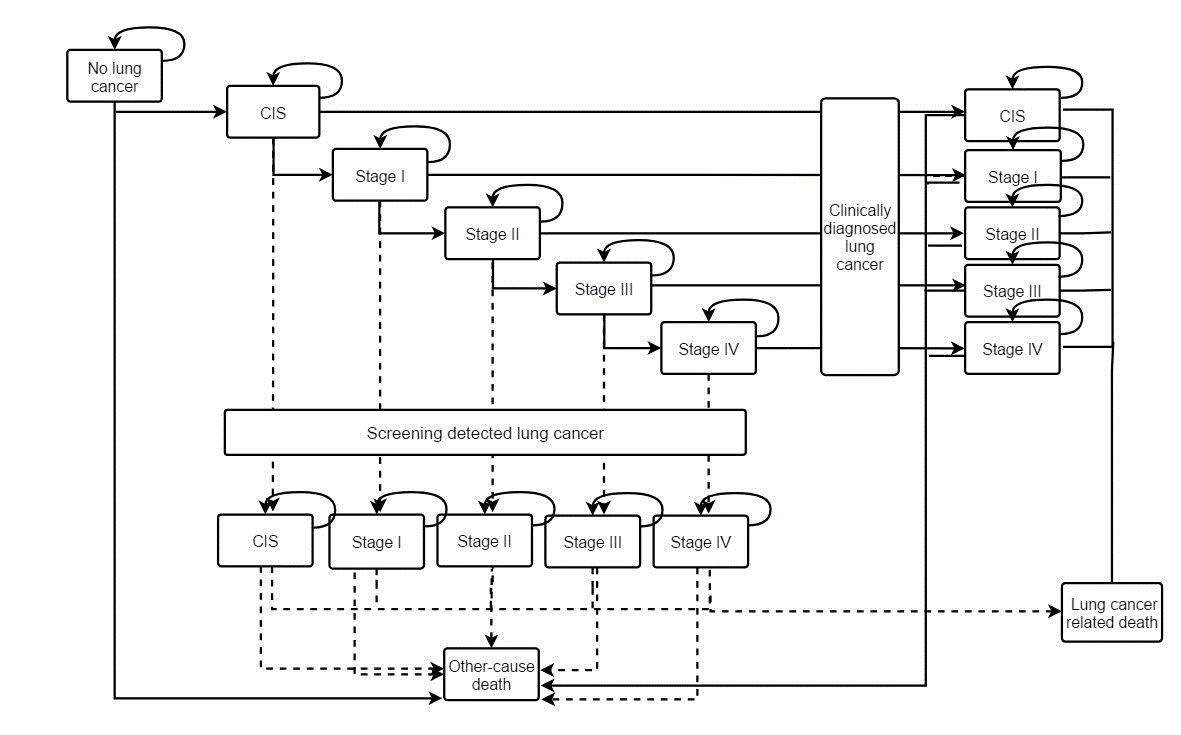


Figure S1 Schematic diagram of Markov model for lung cancer screening

1.1 Parameters in the natural history model

In each cycle, the probability of metastasis from healthy to different stages of lung cancer was the proportion of incidence rate and the corresponding proportion for different clinical stage. According to a national multicenter lung cancer epidemiological survey, the constituent ratios of carcinoma in situ and invasive carcinoma were 0, 0.190, 0.165, 0.346, 0.299^[1]^ respectively. The incidence rate parameters are from the China cancer registry annual report ^[2]^ in 2018. The mortality rate of lung cancer is from the survival analysis of Shanghai population ^[3]^. Since the prognosis of lung cancer in situ is better after surgery, the mortality rate of CIS is set to 0. The mortality of lung cancer removal is obtained by subtracting the age-specific lung cancer mortality from the age-specific all-cause mortality published in the national demographic annual report ^[2,4]^. The initial probability parameters are shown in Table S1. In CIS and stage I invasive carcinoma will progressing to the next stage in a row. In addition to, the probability of metastasis is derived from a German lung cancer screening model and adjusted for ^[5]^ according to the incidence rate in China and Germany. According to a global review, the 1-year metastasis probability of carcinoma in situ developing to stage I lung cancer is estimated to be 0.098 ^[6]^, and the specific metastasis probability parameters are shown in Table S2.

Table S1 Initial and death probability of natural history model

| Age | Incidence | Mortality | | | | All-cause mortality(except lung cancer) |
| --- | --- | --- | --- | --- | --- | --- |
|  |  | **Stage I** | **Stage II** | **Stage III** | **Stage IV** |  |
| 0-5 | 0 | 0.02 | 0.03 | 0.06 | 0.07 | 0.001289 |
| 6-9 | 0 | 0.02 | 0.03 | 0.06 | 0.07 | 0.000299 |
| 10-14 | 0 | 0.02 | 0.03 | 0.06 | 0.07 | 0.000299 |
| 15-19 | 3.2*10^(-6) | 0.02 | 0.03 | 0.06 | 0.07 | 0.000388 |
| 20-24 | 1.29*10^(-5) | 0.02 | 0.03 | 0.06 | 0.07 | 0.000498 |
| 25-29 | 3.5*10^(-5) | 0.02 | 0.03 | 0.06 | 0.07 | 0.000607 |
| 30-34 | 7.34*10^(-5) | 0.02 | 0.03 | 0.06 | 0.07 | 0.000807 |
| 35-39 | 0.000145 | 0.03 | 0.05 | 0.1 | 0.13 | 0.001148 |
| 40-44 | 0.000256 | 0.03 | 0.05 | 0.1 | 0.13 | 0.001712 |
| 45-49 | 0.000466 | 0.04 | 0.07 | 0.1 | 0.18 | 0.002496 |
| 50-54 | 0.000853 | 0.04 | 0.07 | 0.13 | 0.18 | 0.003884 |
| 55-59 | 0.001377 | 0.04 | 0.07 | 0.13 | 0.18 | 0.005645 |
| 60-64 | 0.002056 | 0.04 | 0.07 | 0.13 | 0.18 | 0.009247 |
| 65-69 | 0.002811 | 0.05 | 0.09 | 0.15 | 0.24 | 0.015554 |
| 70-74 | 0.003667 | 0.05 | 0.09 | 0.15 | 0.24 | 0.027761 |
| 75-79 | 0.004058 | 0.06 | 0.13 | 0.27 | 0.35 | 0.044858 |
| 80-84 | 0.003883 | 0.06 | 0.13 | 0.27 | 0.35 | 0.077317 |
| Source | [2] | [3] | [3] | [3] | [3] | [4] |

Table S2 Transition probabilities in natural history model for lung cancer

| **Start state** | **Terminal state** | **Transition probabilities** | **Source** |
| --- | --- | --- | --- |
| CIS | I | 0.0980 | [6] |
| I | II | 0.3682 | [5] |
| I | III | 0.0328 | [5] |
| I | IV | 0.0745 | [5] |
| II | III | 0.2260 | [5] |
| II | IV | 0.1510 | [5] |
| III | IV | 0.1455 | [5] |
| CIS | dead | 0 | [5] |
| I | dead | 0.04 | [5] |
| II | dead | 0.07 | [5] |
| III | dead | 0.13 | [5] |
| IV | dead | 0.18 | [3] |

1.2 Data source of validity evaluation indicators

This work intends to build a natural history model of lung cancer suitable for Chinese population. Therefore, when determining the target parameters of model debugging, priority should be given to the publicly reported data of population-specific epidemiology in China, supplemented by the data reported in a single literature when the data are not available. The commissioning objectives include lung cancer incidence (mortality) rate, age standardized incidence (mortality) rate, cumulative incidence (mortality) rate, life expectancy, proportion of different clinical stages, etc. see Table S1 for specific indicators and their sources.

The parameters related to morbidity and mortality are derived from the relevant data of annual report for cancer registration during 2018-2020 ^[7-9]^. Since 2008, China has initiated a national cancer registration project and gradually carried out population-based information collection on cancer incidence, death and survival in 31 provinces (municipalities directly under the central government and autonomous regions). Among them, the 2018 annual cancer registration report included data from 501 cancer registries across the country, covering a population of 388 million.

The proportion of different clinical stages of lung cancer in natural state comes from a national multicenter clinical epidemiological survey of lung cancer. The survey systematically reviewed the clinical data of 7184 patients with lung cancer from 2005 to 2014, including the composition ratio of clinical stages ^[10]^.

Table S3 Validity indicators and sources

| **Indicator** | **Time range** | **Data source** | |
| --- | --- | --- | --- |
| Incidence（Mortality）crude rate | 2018-2020 | | China Cancer Registration Annual Report |
| Age-standardized incidence（mortality）rate | 2018-2020 | |  |
| Accumulated incidence（mortality）rate | 2018-2020 | |  |
| Proportion of lung cancer | 2005-2014 | | A multicenter clinical epidemiological survey |
| Life expectancy | 2016-2018 | | China Health Statistics Yearbook |
| Age-specific life expectancy | 2017-2019 | | Global Burden of Disease Study（GBD） |

1.3 Calculation method for validity indicators

①Lung cancer incidence and mortality

The incidence (mortality) rate, is so-called the crude incidence (mortality) rate, is the ratio of the number of newly diagnosed cases (deaths) of lung cancer to the corresponding population.

Incidence（mortality）rate per 100 000=$\frac{new cases\left（ new cancer deaths \right）}{population at the same period}*100000$

Since the crude incidence (mortality) rate is greatly affected by the age structure of the population, in order to ensure the comparability between the model output results and the cancer registration data, it is necessary to calculate the age standardized incidence (mortality) rate according to the age structure of a standard population. This work uses the population composition of the fifth national census in 2000 and Segi's world standard population to calculate the China standard rate and world standard rate respectively. The calculation formula is:

Age-standardization incidence(mortality) rate per 100000 =$\frac{\sum standard population in corresponding age group*age-specific rate}{\sum standard population}$

In addition to the age standardized rate, the cumulative incidence (mortality) rate can also eliminate the influence of age structure and can be used for the comparison of different incidence (mortality) data. Specifically, it refers to the total indicator of cumulative incidence (mortality) rate according to age in a certain age stage. This work selects the cumulative incidence (mortality) rate aged 0-74, the calculation formula is:

Cumulative incidence (mortality) rate（%）=（$\sum（age-specific incidence\left（ \mathrm{mortality} \right）rate*age group））*100$

Table S4 Standard population of China and Segi’s population

| **Age group（years）** | **China Standard population (2000)** | **Segi’s population** |
| --- | --- | --- |
| 0~ | 13793799 | 2400 |
| 1~ | 55184575 | 9600 |
| 5~ | 90152587 | 10000 |
| 10~ | 125396633 | 9000 |
| 15~ | 103031165 | 9000 |
| 20~ | 94573174 | 8000 |
| 25~ | 117602265 | 8000 |
| 30~ | 127314298 | 6000 |
| 35~ | 109147295 | 6000 |
| 40~ | 81242945 | 6000 |
| 45~ | 85521045 | 6000 |
| 50~ | 63304200 | 5000 |
| 55~ | 46370375 | 4000 |
| 60~ | 41703848 | 4000 |
| 65~ | 34780460 | 3000 |
| 70~ | 25574149 | 2000 |
| 75~ | 15928330 | 1000 |
| 80~ | 7989158 | 500 |
| 85+ | 4001925 | 500 |
| Total | 1242612226 | 100000 |

② Proportion for clinical stages

The proportion for clinical stages indicates how much the number of stage-specific new cancer cases accounts for the number of all the new lung cancer cases. The formula is:

Proportion for clinical stages(%)=$\frac{No. of cases of a particular stage}{No. of cases of all stages}$

③ Life expectancy

Life expectancy refers to taking the age-specific mortality rate of a certain year as a fixed value, assuming that it does not change with time, and estimating the average time that people born in a certain period can survive, usually in "years". This work simulates a group of 0-year-old birth cohort, and plans to use the life table method to calculate the life expectancy. The specific calculation formula is as follows:

Age-specific mortality $m_{x}=\frac{Number of deaths during the year}{Average annual population}$

Age-specific dead probability $q_{x}=\frac{2nm_{x}}{2+nm_{x}}$ ，n represents years

Age-specific survival probability $p_{x}={1-q}_{x}$

Relations between age-specific survive population$l_{x}$ and dead population $d_{x}$

$$d_{x}=l_{x}q_{x}$$

$$l_{x+n}=l_{x}-d_{x}$$

Relations between life years $L_{x}$ and total life years $T_{x}$

$$L_{X}=n\frac{l_{x}+l_{x+n}}{2}$$

$$T_{x}=\sum L_{x}$$

- 1. Validity evaluation of natural history model

① Incidence and mortality

Using the natural history model constructed above to simulate a birth cohort of 1000 people, the results show that the crude incidence and mortality rates of lung cancer are 64.17/100000 and 55.83/100000 respectively, which are similar to the data of China annual cancer registration reports from 2018 to 2020; After standardization by China's population composition, the rate is 37.2/100000, and the rate after the standardization of Segi's world population composition is 36.98/100000, which is basically consistent with the data of 2018-2020 National Cancer Registration annual report; The cumulative incidence rate of 0-74 years by model simulation is basically consistent with the annual data of tumor registration. Compared with the annual data of national cancer registration, the simulation value of mortality is a little bit higher. See Table S5 for details.

Table S5 Validity indicator: incidence and mortality of lung cancer

| **Indicator** | **Simulated value** | **Observed value(Cancer registration report）** | | |
| --- | --- | --- | --- | --- |
|  |  | **2015** | **2016** | **2017** |
| Incidence |  |  |  |  |
| Crude rate /100000 | 64.17 | 58.91 | 60.04 | 74.87 |
| ASR China /100000 | 37.20 | 35.57 | 36.04 | 39.23 |
| ASR World /100000 | 36.98 | 35.54 | 36.02 | 47.58 |
| 0-74 cumulative rate(%) | 4.73 | 4.34 | 4.41 | 5.88 |
| Mortality |  |  |  |  |
| Crude rate /100000 | 55.83 | 47.79 | 48.42 | 27.85 |
| ASR China /100000 | 28.78 | 27.99 | 27.95 | 26.24 |
| ASR World /100000 | 28.58 | 27.85 | 27.87 | 26.10 |
| 0-74 cumulative rate(%) | 3.64 | 3.27 | 3.27 | 3.01 |

② Proportion for clinical stages


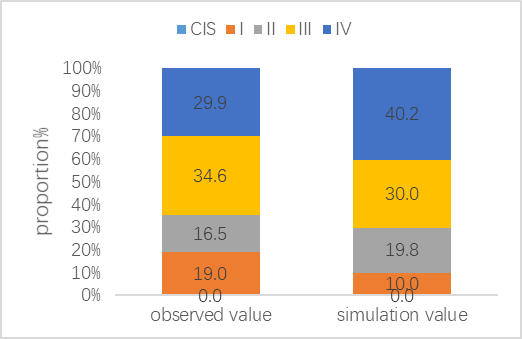


Figure S2 Proportion for clinical stages

③ Life expectancy

The model simulation reveals that the life expectancy of China's birth cohort is 76.8 years, which is consistent with the observed values (76.5, 76.7 and 77.0) reported from the 2016-2018 China Health Statistical Yearbook ^[11]^, and is consistent with the observed value in GBD for 2018, which is 77.4 (95% uncertainty interval: 76.2-78.8) years ^[12]^. The trend of life expectancy corresponding to different ages is basically consistent with the observed value of GBD.


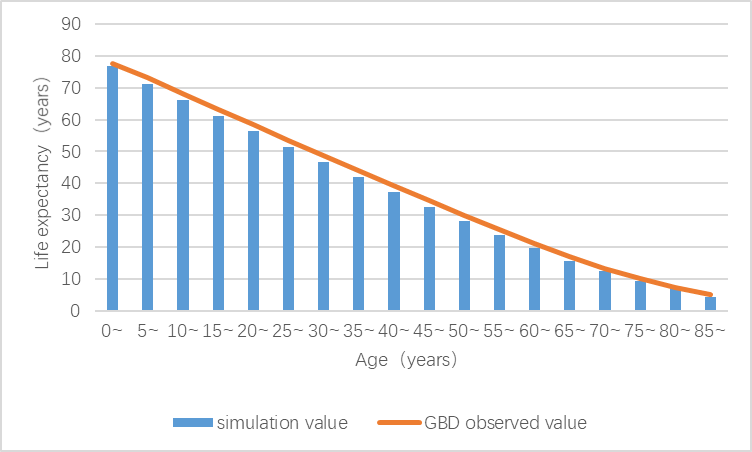


Figure S3 Comparison between GBD observed value and simulation value in life expectancy

**References**

[1] Shi JF, Wang L, Wu N, et al, Clinical characteristics and medical service utilization of lung cancer in China, 2005–2014: Overall design and results from a multicenter retrospective epidemiologic survey, Lung Cancer,2019(128)91-100, doi:10.1016/j.lungcan.2018.11.031.

[2] He, J. and W. Chen, CHINA CANCER REGISTRY ANNUAL REPORT (2018). 2019: People's Medical Publishing House.ISBN:978-7-117-28585-8.

[3] Zhang, M., et al., Survival analysis of patients with lung cancer in Shanghai. China Oncology, 2017.

[4] Tabulation on the 2010 Population Census of the People's Republic of China. 2010, Department of Population and Employment Statistics of the National Bureau of Statistics of China: Beijing, China.

[5] Hofer F, Kauczor HU, Stargardt T. Cost-utility analysis of a potential lung cancer screening program for a high-risk population in Germany: A modelling approach. Lung Cancer. 2018 Oct;124:189-198.doi:10.1016/j.lungcan.2018.07.036.Epub 2018 Jul 24. https://doi.org/10.1016/j.lungcan.2018.07.036

[6] Anindo K. Banerjee. Preinvasive Lesions of the Bronchus,Journal of Thoracic Oncology, 2009.4(4),Pages 545-551.doi:10.1097/JTO.0b013e31819667bd.

[7] National Cancer Institute. 2018 China cancer registry annual report.2018. People's Medical Publishing House, Beijing, China.

[8] National Cancer Institute. 2019 China cancer registry annual report.2019. People's Medical Publishing House, Beijing, China.

[9] National Cancer Institute. 2020 China cancer registry annual report.2020. People's Medical Publishing House, Beijing, China.

[10] Shi JF, Wang L, Wu N, et al, Clinical characteristics and medical service utilization of lung cancer in China, 2005–2014: Overall design and results from a multicenter retrospective epidemiologic survey, Lung Cancer,2019(128)91-100, doi:10.1016/j.lungcan.2018.11.031.

[11] National Health Commission (2018). 2018 China Health Statistical Yearbook Beijing: China Union Medical College Press, 231-239

[12] Institute for health metrics and evaluation (2019).GBD Compare.2019.2021-12-30.Available at:http://vizhub.healthdata.org/gbd-cpmpare/.

**Appendix 2. Scenario analysis**

Table S6 Outcomes of scenario analysis with diverse compliance rates

| **Start age** | **Strategies** | **Baseline compliance rate** | | **LDCT 95%** | | **PRS 90%** | |
| --- | --- | --- | --- | --- | --- | --- | --- |
|  |  | **ICER** | **ICUR** | **ICER** | **ICUR** | **ICER** | **ICUR** |
| 50 | #1 LDCT | 174100.78 | 147955.43 | 174245.37 | 148078.31 | 174100.78 | 147955.43 |
|  | #2 PRS&LDCT | 338167.54 | 287284.80 | 338131.39 | 287271.07 | 338845.14 | 287890.94 |
| 55 | #1 LDCT | 141232.37 | 119234.89 | 141381.92 | 119361.15 | 141232.37 | 119234.89 |
|  | #2 PRS&LDCT | 290527.78 | 245200.98 | 290415.18 | 245119.31 | 291027.85 | 245647.09 |
| 60 | #1 LDCT | 116653.80 | 97725.45 | 116810.94 | 97857.09 | 116653.80 | 97725.45 |
|  | #2 PRS&LDCT | 263601.61 | 220631.04 | 263151.34 | 220288.15 | 263555.16 | 220653.84 |
| 65 | #1 LDCT | 106141.26 | 86260.84 | 106319.32 | 86405.54 | 106141.26 | 86260.84 |
|  | #2 PRS&LDCT | 281857.05 | 228519.15 | 280756.90 | 227714.83 | 280947.54 | 227936.81 |
| 70 | #1 LDCT | 107958.13 | 82867.31 | 108186.94 | 83042.94 | 107958.13 | 82867.31 |
|  | #2 PRS&LDCT | 389353.97 | 297098.38 | 386217.27 | 294987.81 | 385517.91 | 294707.47 |

**Appendix 3. CHEERS Checklist
Items to include when reporting economic evaluations of health interventions**

The **ISPOR CHEERS Task Force Report**, Consolidated Health Economic Evaluation Reporting Standards (CHEERS)—Explanation and Elaboration: A Report of the ISPOR Health Economic Evaluations Publication Guidelines Good Reporting Practices Task Force, provides examples and further discussion of the 24-item CHEERS Checklist and the CHEERS Statement. It may be accessed via the Value in Health or via the ISPOR Health Economic Evaluation Publication Guidelines – CHEERS: Good Reporting Practices webpage: http://www.ispor.org/TaskForces/EconomicPubGuidelines.asp

| **Section/item** | **Item No** | **Recommendation** | **Reported in section (paragraph no)** |
| --- | --- | --- | --- |
| **Title and abstract** |  |  |  |
| Title | 1 | Identify the study as an economic evaluation or use more specific terms such as ‘cost-effectiveness analysis’, and describe the interventions compared. | Title |
| Abstract | 2 | Provide a structured summary of objectives, perspective, setting, methods (including study design and inputs), results (including base case and uncertainty analyses), and conclusions. | Abstract |
|  |  |  |  |
| **Introduction** |  |  |  |
| Background and objectives | 3 | Provide an explicit statement of the broader context for the study.  Present the study question and its relevance for health policy of practice decisions. | Introduction |
| **Methods** |  |  |  |
| Target population and subgroups | 4 | Describe characteristics of the base case population and subgroups analysed, including why they were chosen. | 2.1 Study design and model description |
| Setting and location | 5 | State relevant aspects of the system(s) in which the decision(s) need(s) to be made. | 2.1 Study design and model description |
| Study perspective | 6 | Describe the perspective of the study and relate this to the costs being evaluated. | 2.1 Study design and model description |
| Comparators | 7 | Describe the interventions or strategies being compared and state why they were chosen. | 2.1 Study design and model description;2.3 Evaluated strategies |
| Time horizon | 8 | State the time horizon(s) over which costs and consequences are being evaluated and say why appropriate. | 2.1 Study design and model description |
| Discount rate | 9 | Report the choice of discount rate(s) used for costs and outcomes and say why appropriate. | 2.1 Study design and model description |
| Choice of health outcomes | 10 | Describe what outcomes were used as the measure(s) of benefit in the evaluation and their relevance for the type of analysis performed. | 2.4 Outcome measures |
| Measurement of effectiveness | 11a | *Single study-based estimates:* Describe fully the design features of the single effectiveness study and why the single study was a sufficient source of clinical effectiveness data. |  |
|  | 11b | *Synthesis-based estimates*: Describe fully the methods used for identification of including studies and synthesis of clinical effectiveness data. | 2.4 Outcome measures |
| Measurement and valuation of preference based outcomes | 12 | If applicable, describe the population and methods used to elicit preferences for outcomes. | Not applicable |
| Estimating resources and costs | 13a | Single study-based economic evaluation: Describe approaches used to estimate resource use associated with the alternative interventions. Describe primary or secondary research methods for valuing each resource item in terms of its unit cost. Describe any adjustments made to approximate to opportunity costs. | Not applicable |
|  | 13b | Model-based economic evaluation: Describe approaches and data sources used to estimate resource use associated with model health states. Describe primary or secondary research methods for valuing each resource item in terms of its unit cost. Describe any adjustments made to approximate to opportunity costs. | 2.2 Model input parameters；Appendix |
| Currency, price date, and conversion | 14 | Report the dates of the estimated resource quantities and unit costs. Describe methods for adjusting estimated unit costs to the year of reported costs if necessary. Describe methods for converting costs into a common currency base and the exchange rate. | 2.2 Model input parameters |
| Choice of model | 15 | Describe and give reasons for the specific type of decision- analytical model used. Providing a figure to show model structure is strongly recommended. | 2.1 Study design and model description; Fig. 1 |
| Assumptions | 16 | Describe all structural or other assumptions underpinning the decision-analytical model. | 2.1 Study design and model description |
| Analytical methods | 17 | Describe all analytical methods supporting the evaluation. This could include methods for dealing with skewed, missing, or censored data; extrapolation methods; methods for pooling data; approaches to validate or make adjustments (such as half cycle corrections) to a model; and methods for handling population heterogeneity and uncertainty. | 2.1 Study design and model description;2.5 Sensitivity analysis and scenario analysis |
| **Results** |  |  |  |
| Study parameters | 18 | Report the values, ranges, references, and, if used, probability distributions for all parameters. Report reasons or sources for distributions used to represent uncertainty where appropriate. Providing a table to show the input values is strongly recommended. | Table 1 |
| Incremental costs and outcomes | 19 | For each intervention, report mean values for the main categories of estimated costs and outcomes of interest, as well as mean differences between the comparator groups. If applicable, report incremental cost-effectiveness ratios. | 3.1 Base-case analysis; Table 3 |
| Characterising uncertainty | 20a | Single study-based economic evaluation: Describe the effects of sampling uncertainty for the estimated incremental cost and incremental effectiveness parameters, together with the impact of methodological assumptions (such as discount rate, study perspective). | Not applicable |
|  | 20b | Model-based economic evaluation: Describe the effects on the results of uncertainty for all input parameters, and uncertainty related to the structure of the model and assumptions. | 3.2 Sensitivity analysis and scenario analysis; Figs 2-3 |
| Characterising heterogeneity | 21 | If applicable, report differences in costs, outcomes, or cost-effectiveness that can be explained by variations between subgroups of patients with different baseline characteristics or other observed variability in effects that are not reducible by more information. | Not applicable |
| **Discussion** |  |  |  |
| Study findings, limitations, generalisability, and current knowledge | 22 | Summarise key study findings and describe how they support the conclusions reached. Discuss limitations and the generalisability of the findings and how the findings fit with current knowledge. | Discussion; Conclusion |
| **Other** |  |  |  |
| Source of funding | 23 | Describe how the study was funded and the role of the funder in the identification, design, conduct, and reporting of the analysis. Describe other non-monetary sources of support. | Funding/Support |
| Conflicts of interest | 24 | Describe any potential for conflict of interest of study contributors in accordance with journal policy. In the absence of a journal policy, we recommend authors comply with International Committee of Medical Journal Editors recommendations. | Conflict of Interest Disclosures |

For consistency, the CHEERS Statement checklist format is based on the format of the CONSORT statement checklist

The ISPOR CHEERS Task Force Report provides examples and further discussion of the 24-item CHEERS Checklist and the CHEERS Statement. It may be accessed via the Value in Health link or via the ISPOR Health Economic Evaluation Publication Guidelines – CHEERS: Good Reporting Practices webpage: http://www.ispor.org/TaskForces/EconomicPubGuidelines.asp

The citation for the CHEERS Task Force Report is:
Husereau D, Drummond M, Petrou S, et al. Consolidated health economic evaluation reporting standards (CHEERS)—Explanation and elaboration: A report of the ISPOR health economic evaluations publication guidelines good reporting practices task force. Value Health 2013;16:231-50.
